# Modulation of nuclear REST by alternative splicing: a potential therapeutic target for Huntington's disease

**DOI:** 10.1111/jcmm.13209

**Published:** 2017-05-19

**Authors:** Guo‐Lin Chen, Qi Ma, Dharmendra Goswami, Jianyu Shang, Gregory M. Miller

**Affiliations:** ^1^ Department of Pharmaceutical Sciences and Center for Drug Discovery School of Pharmacy Northeastern University Boston MA USA; ^2^ Guangxi Collaborative Innovation Center for Biomedicine Guangxi Medical University Nanning Guangxi China; ^3^ Research Center for Regenerative Medicine of Guangxi Guangxi Medical University Nanning Guangxi China; ^4^ Department of Psychiatry Institute for Human Performance SUNY Upstate Medical University Syracuse NY USA; ^5^ Center for the Study of Traumatic Encephalopathy Boston University School of Medicine Boston MA USA; ^6^ Department of Neurology Boston University School of Medicine Boston MA USA; ^7^ VA Boston HealthCare System Boston MA USA; ^8^ Department of Chemical Engineering School of Engineering Northeastern University Boston MA USA

**Keywords:** REST/NRSF, alternative splicing, nuclear translocation, gene therapy, Huntington's disease, antisense oligos, Syn‐1, Stmn2, PPARγ

## Abstract

Huntington's disease (HD) is caused by a genetically mutated huntingtin (mHtt) protein with expanded polyQ stretch, which impairs cytosolic sequestration of the repressor element‐1 silencing transcription factor (REST), resulting in excessive nuclear REST and subsequent repression of neuronal genes. We recently demonstrated that *REST* undergoes extensive, context‐dependent alternative splicing, of which exon‐3 skipping (∆E_3_)—a common event in human and nonhuman primates—causes loss of a motif critical for REST nuclear targeting. This study aimed to determine whether ∆E_3_ can be targeted to reduce nuclear REST and rescue neuronal gene expression in mouse striatal‐derived, mHtt‐expressing STHdh^Q111/Q111^ cells—a well‐established cellular model of HD. We designed two morpholino antisense oligos (ASOs) targeting the splice sites of *Rest* E_3_ and examined their effects on ∆E_3_, nuclear Rest accumulation and Rest‐controlled gene expression in STHdh^Q111/Q111^ cells. We found that (1) the ASOs treatment significantly induced ∆E_3_, reduced nuclear Rest, and rescued transcription and/or mis‐splicing of specific neuronal genes (*e.g. Syn1* and *Stmn2*) in STHdh^Q111/Q111^ cells; and (2) the ASOs‐induced transcriptional regulation was dependent on ∆E_3_ induction and mimicked by siRNA‐mediated knock‐down of *Rest* expression. Our findings demonstrate modulation of nuclear REST by ∆E_3_ and its potential as a new therapeutic target for HD and provide new insights into environmental regulation of genome function and pathogenesis of HD. As ∆E_3_ is modulated by cellular signalling and linked to various types of cancer, we anticipate that ∆E_3_ contributes to environmentally tuned REST function and may have a broad range of clinical implications.

## Introduction

Originally identified as a transcriptional repressor of neuronal genes, the repressor element‐1 silencing transcription factor (REST, also named NRSF for neuron‐restrictive silencing factor) is now recognized as a coordinate transcriptional and epigenetic regulator that orchestrates cellular epigenome 1, 2. REST contains a DNA‐binding domain consisting of eight zinc fingers (ZFs), which is sandwiched by two repression domains capable of recruiting numerous transcriptional and epigenetic cofactors, through which REST promotes dynamic, context‐dependent chromatin remodelling and repression or activation of thousands of genes 2, 3. As such, REST controls many cellular processes fundamental to normal physiology and pathological conditions and is implicated in a wide range of human diseases including cancer and neurodegenerative diseases.

The physical separation of the genome from cytoplasm by nuclear envelope in eukaryotic cells requires translocation of REST from cytoplasm to nucleus to modulate genome function. It was documented that ZF‐5 is critical for REST nuclear targeting 4, 5 and that altered nuclear REST is implicated in adenovirus‐induced cell transformation 6, ageing and neurodegenerative diseases including Huntington's and Alzheimer's diseases (HD and AD) 7, 8. Notably, a protein named huntingtin (Htt) associates with REST through a cytoskeletal complex which prevents nuclear translocation of REST; however, Htt is mutated in HD, leading to impaired cytosolic sequestration of REST and excessive accumulation of nuclear REST, which in turn represses neuronal genes important for the maintenance and function of specific neurons 8, 9, 10. Accordingly, rescue of neuronal gene expression through modulation of REST activity has been suggested as a therapeutic strategy for HD 11, 12.

As a multi‐exonic gene, *REST* undergoes alternative splicing, a cotranscriptional process which enables a single gene to produce multiple mRNA and protein variants, with an N‐terminal REST4 isoform having been well documented 8, 13. Recently, we identified 45 *REST* mRNA splice variants with highly context‐dependent expression, suggesting an underappreciated role of alternative splicing in modulation of REST function 14. Particularly, skipping of exon‐3 (∆E_3_), a common splicing event which eliminates ZF‐5 critical for REST nuclear targeting, is linked to cancer and modulated by pioglitazone—a highly selective activator of the peroxisome proliferator‐activated receptor gamma (PPARγ) exerting biological actions overlapping with REST 15, 16, 17. Hence, we suggested that ∆E_3_ may act as an endogenous, manipulable modulator of REST activity, and it may be targeted to treat HD related to REST dysfunction.

To test this hypothesis, we determined whether manipulation of ∆E_3_ alters nuclear REST and neuronal gene expression in a cell model of HD. We designed two antisense oligos (ASOs) targeting the splice sites of *Rest* E_3_ and examined their effects on ∆E_3_, nuclear Rest and neuronal gene expression in STHdh^Q111/Q111^ cells. We demonstrated that treatment of STHdh^Q111/Q111^ cells with the ASOs significantly induced ∆E_3_, reduced nuclear Rest and rescued transcription and/or mis‐splicing of specific neuronal genes and that the ASOs‐induced transcriptional regulation was dependent on ∆E_3_ induction while mimicked by siRNA knock‐down of Rest. Our findings validate ∆E_3_ as a modulator of nuclear REST and a potential therapeutic target for HD and provide new insights into HD pathogenesis.

## Materials and methods

### Cell culture and treatment

STHdh^Q7/7^ and STHdh^Q111/111^ cells (Coriell Institute, Camden, NJ, USA) were cultured in advanced DMEM (Invitrogen, Carlsbad, CA, USA) supplemented with 10% heat‐inactivated FBS, 1% penicillin/streptomycin and 200 μg/ml G418 (Invitrogen) at 33°C in a 5% CO_2_ incubator. The two cell lines were established from knock‐in transgenic mice containing humanized *Htt* exon 1 with seven and 111 polyQ repeats, respectively, of which STHdh^Q111/Q111^ is a well‐established cell model of HD 18, 19, 20, 21. Passages 3‐9 of the cells were used for all experiments.

For ASOs treatment, 3 μM of morpholino ASOs (I_2_E_3_ and E_3_I_3_, alone or combined) or a control oligo (Gene Tools, Philomath, OR, USA) were delivered into cells using 6 μM of Endo‐Porter (Gene Tools). For RNAi experiment, 10 nM of 27mer Rest (mouse) siRNAs (siRNA‐1 and ‐2) or a negative siRNA (OriGene, Rockville, MD, USA) were transfected into cells using siTran 1.0 Transfection Reagent (OriGene). Cells were harvested for various analyses at 48 hrs post‐treatment, and experiments were performed in duplicate on three independent occasions.

### Immunofluorescence confocal microscopy and image analysis

Two widely used anti‐REST antibodies, sc‐25398 (Santa Cruz, Dallas, TX, USA) and ab21635 (Abcam, Cambridge, MA, USA), were employed to perform immunocytochemistry (ICC). Briefly, cells cultured on poly‐D‐lysine‐coated coverslips were treated with ASOs (I_2_E_3_ + E_3_I_3_) or a control oligo. After 48‐hr incubation, cells were fixed with 4% paraformaldehyde, permeabilized with 0.3% Triton X‐100 and incubated with sc‐25398 (1:100) or ab21635 (1:200), followed by incubation with a goat anti‐rabbit secondary antibody conjugated with Alexa dye (1:500; Invitrogen). Nuclei were stained with Hoechst‐33342 (Thermo Scientific, Waltham, MA, USA), and cells were mounted on glass slides.

For confocal microscopy, image stacks along the *z*‐axis were acquired using a Leica TCS SP5 Spectral Confocal Microscope (Leica Microsystems, Cambridge, UK). Image acquisition settings were kept the same for all scans in the same experiment group when fluorescence intensity was compared. Images were analysed using ImageJ program (NIH). Fluorescence intensities measured as integrated pixel intensities were determined for the entire cell and its nucleus area, respectively. Nucleus percentage of fluorescence intensity was calculated for each cell, and values of 100 cells in each group were averaged and presented as Mean ± S.E.M. All groups to be compared were run simultaneously using cells from the same culture preparations.

### RNA isolation and cDNA synthesis

Total RNA was extracted using Trizol^®^ reagent (Invitrogen) and reverse transcribed into cDNA using Superscript™ III reverse transcriptase and oligo‐dTs (Invitrogen). To avoid DNA contamination, samples were treated with RQ1 RNase‐free DNase I (Promega, Madison, WI, USA) for 1 hr at 37°C. Synthesized cDNA was diluted to 50 ng/μl for use.

### Polymerase chain reaction (PCR)

For detection of ∆E_3_, nested PCR was performed as previously described 14 in a MJ Research PTC‐200 Peltier Thermal Cycler using Rest‐E_1_F_2_/E_4_R_3_ and Rest‐E_2_F_2_/E_4_R_1_ (or ‐E_1_F_1_/E_4_R_1_) (Table 1) as the primer set for 1st‐ and 2nd‐round amplification, respectively. PCR with Syn1‐E_7_F_1_/E_9_R_1_ was performed to verify Syn1 variants with/‐out intron‐8 (I_8_). PCR products were electrophoresed on 2% agarose gel, excised, purified and sequence verified. DNA sequencing was serviced by Functional Biosciences (Madison, WI, USA).

**Table 1:**
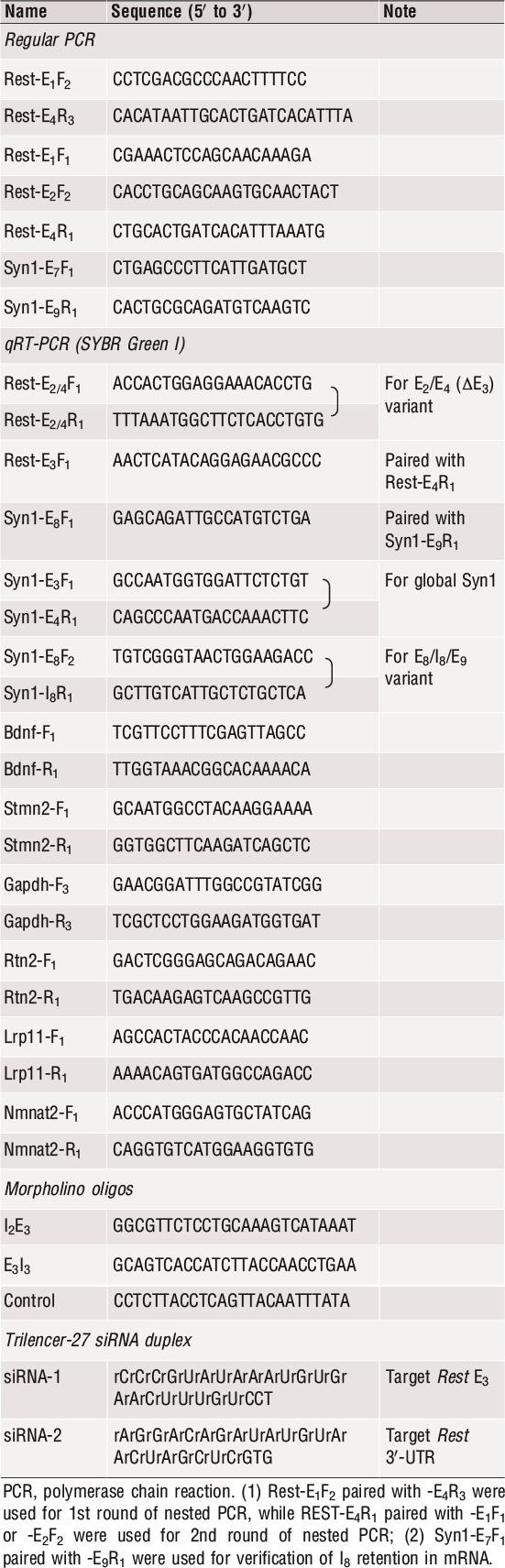
Oligos used for PCR, ∆E_3_ manipulation and RNAi

### Quantitative real‐time PCR (qRT‐PCR)

SYBR Green qRT‐PCR for *Bdnf*,* Stmn2*,* Syn1*,* Gapdh, Nmnat2, Lrp11, Rtn2* and specific *Rest* variants was performed using primers listed in Table 1 in a Roche LightCycler 2.0 system (Roche Diagnostics, Indianapolis, IN, USA) as previously described 14. The unique amplicon was verified for each assay, and the threshold cycle (C_t_) values were used to evaluate expression change between groups using the 2^−ΔΔCt^ approach 22 with *Gapdh* as the reference.

### Transcriptional profiling

Three total RNA samples of high quality (RNA integrity number: 8.6–9.4) isolated from cells treated with ASOs (I_2_E_3_ + E_3_I_3_) (Q111‐ASOs) or a control oligo (Q111‐ and Q7‐Control) were transcriptionally profiled by the Affymetrix's GeneChip^®^ Mouse Gene 2.0 ST Array serviced by EpigenDx (Hopkinton, MA, USA). Pairwise comparisons of expression between the samples were performed by analysis of variance (anova) using the Partek^®^ Genomic Suite^®^ software (St. Louis, MO, USA). The heat map was generated by hierarchical clustering of expression values using the genes generated by anova comparisons between the samples. The mean expression is shifted to 0 and scaled to a S.D. of 1.

### Western blotting for Stmn2

Cytoplasmic protein fraction was extracted using NE‐PER^®^ Nuclear and Cytoplasmic extraction reagents (Thermo Scientific), and protein concentration was determined by Bradford assay. The same amount (40 μg) of protein was boiled with 2× Laemmli buffer, electrophoresed on a 10% SDS‐PAGE gel and electrotranslocated onto an Immun‐Blot PVDF membrane (Bio‐Rad, Hercules, CA, USA) presoaked in methanol. The membrane was blocked with 5% non‐fat milk and incubated with a goat anti‐STMN2 (SAB2500997) (Sigma‐Aldrich, St Louis, MO, USA) at 1:500 overnight at 4°C, followed by incubation with a rabbit anti‐goat IgG (A5420; Sigma‐Aldrich) at 1:2500 for 1 hr at room temperature. Gapdh was blotted as a loading control using a mouse anti‐GAPDH (TA802519; OriGene) and a rabbit antimouse IgG (A9044; Sigma‐Aldrich). Immunoreactive signals were detected using the VisiGlo™ Select HRP Chemiluminescent Substrate Kit (Amresco, Solon, OH, USA) with an ECL‐based LAS‐3000 image system (Fujifilm, Life Science, New Haven, CT, USA). Densitometric analysis was carried out using ImageGauge (Fujifilm). Assays were performed on three independent occasions.

### ELISA for Bdnf

Cells were lysed using the lysis buffer consisting of 10% glycerol, 25 mM Tris‐HCl (pH 7.5), 150 mM NaCl, 1% Triton X‐100, 5 mM EDTA and 1 mM EGTA supplemented with 1:100 Halt™ Protease Inhibitor cocktail (Thermo Scientific). Samples were homogenized, sonicated and centrifuged at maximum speed for 10 min. at 4°C, and the supernatants were collected and stored at −80°C. Samples were assayed using a mouse Bdnf ELISA Kit (Boster, Fremont, CA, USA). Assays were performed in triple on three independent occasions.

### Bioinformatics and data analysis

Bioinformatics were performed by checking specific tracks provided by the UCSC Genome Browser (http://genome.ucsc.edu). Statistics were carried out using the SAS Software Version 9.4 (SAS Institute Inc., Cary, NC, USA). Comparisons of nuclear REST percentage and mRNA/protein expression level between different groups were performed by appropriate anova or Student's *t‐*test.

## Results

### Induction of ∆E_3_ by ASOs targeting the splice sites of E_3_

**Figure 1:**
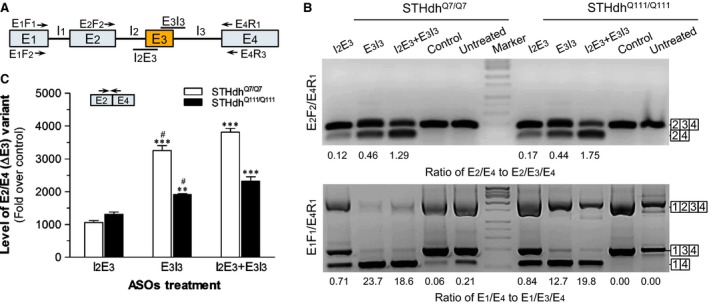
Induction of *Rest* ∆E_3_ by specific ASOs in STHdh^Q7/Q7^ and STHdh^Q111/Q111^ cells. (**A**) Schematic structure of Rest gene and targeted splicing sites of the two ASOs (I_2_E_3_ and E_3_I_3_). Primers used for polymerase chain reaction (PCR) detection of ∆E_3_ are shown by arrows. (**B**) PCR verification of ASOs‐induced ∆E_3_. Cells treated with the ASOs (I_2_E_3_ and/or E_3_I_3_, 3 μM of each) or a control oligo were harvested for RNA isolation at 48 hrs post‐treatment. Nested PCR was performed with E_1_F_1_/E_4_R_3_ and E_2_F_2_/E_4_R_1_ (or E_1_F_1_/E_4_R_1_) as the primer set for the 1st‐ and 2nd‐round amplification, respectively. Amplicons were sequence verified, and ratio of the variants with/‐out ∆E_3_ for each lane was analysed by GeneTools software (Syngene, Cambridge, UK). (**C**) Relative expression of the E_2_/E_4_ (∆E_3_) mRNA assayed by qRT‐PCR with E_2/4_F_1_/R_1_. The E_2_/E_4_ mRNA levels were expressed as folds over the control using *Gapdh* as the reference. Data are shown as Mean ± S.E.M. anova:* F* = 165.54, *P* < 0.0001 for STHdh^Q7/Q7^; *F* = 32.76, *P* = 0.0006 for STHdh^Q111/Q111^. **P < 0.01, ***P < 0.001 compared with I_2_E_3_ group; ^#^ *P* < 0.05 compared with I_2_E_3_ + E_3_I_3_ group.

We designed two ASOs (I_2_E_3_ and E_3_I_3_, Fig. 1A) targeting the splice acceptor and donor sites of *Rest* E_3_, respectively. As shown in Figure 1B, both ASOs induced ∆E_3_ in STHdh^Q7/Q7^ and STHdh^Q111/Q111^ cells, as indicated by formation and up‐regulation of the E_2_/E_4_ (∆E_3_) and E_1_/E_4_ (∆E_2_ + ∆E_3_) variants, respectively, as well as apparent reduction of E_3_‐retained variants (E_2_/E_3_/E_4_ and E_1_/E_3_/E_4_). In both STHdh^Q7/Q7^ and STHdh^Q111/Q111^ cells, based on the ratio of variants with/‐out ∆E_3_, E_3_I_3_ induced more ∆E_3_ than I_2_E_3_, while combination of the two ASOs yielded the most ∆E_3_ induction, such is validated by qRT‐PCR‐assayed expression of the E_2_/E_4_ variant (Fig. 1C) using primer set *Rest*‐E_2/4_F_1_/R_1_ listed in Table 1. The ASOs also induced ∆E_3_ in mouse primary hippocampal neurons (implying a potential *in vivo* effect), along with cells of other species including human NCCIT and rat RN46A cells (Fig. S1A), which may be explained by the highly conserved intron–exon junctions of E_3_ as indicated by the ‘Vertebrate Multiz Alignment & Conservation’ track from the UCSC Genome Browser (data not shown).

### ASOs‐induced reduction of nuclear Rest in STHdh^Q111/Q111^ cells

**Figure 2:**
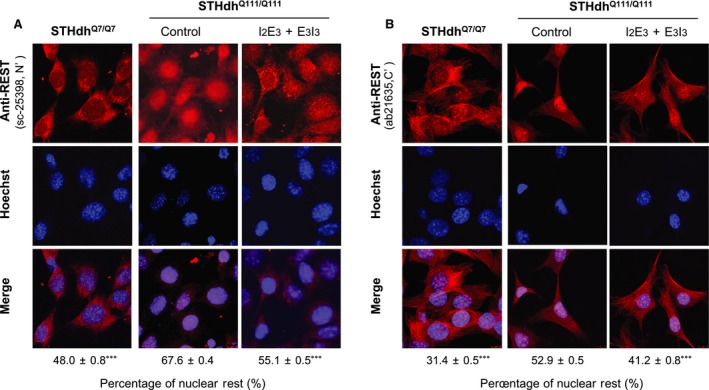
Immunofluorescence analysis of STHdh^Q7/Q7^ and STHdh^Q111/Q111^ cells with or without ASOs treatment. ICC was performed on with P3 and P6 cells using the antibody sc‐25398 **(A)** and ab21635 **(B)** against N‐ and C‐terminal of REST, respectively. Note that the two ASOs were combined for the treatment. Percentage of nuclear REST was analysed by ImageJ, and values of 100 cells were averaged for each group and shown as Mean ± S.E.M. ***P < 0.001 compared with the control STHdh^Q111/Q111^ group by Student's *t*‐test.

To examine whether the ASOs reduced nuclear Rest in STHdh^Q111/Q111^ cells, we performed ICC with two antibodies (sc‐25398 and ab21635) against the N‐ and C‐terminal of REST, respectively. As shown in Figure 2 and Figure S2, regardless of the antibody, accumulation of nuclear Rest was significantly higher in STHdh^Q111/Q111^ than that in STHdh^Q7/Q7^, while ASOs treatment (I_2_E_3_ + E_3_I_3_) significantly reduced nuclear Rest in STHdh^Q111/Q111^ cells. ASOs‐induced reduction in nuclear Rest was also observed in RN46A as indicated by ICC (Fig. S1B).

### ASOs‐induced transcriptional derepression of Rest target genes in STHdh^Q111/Q111^ cells

**Figure 3:**
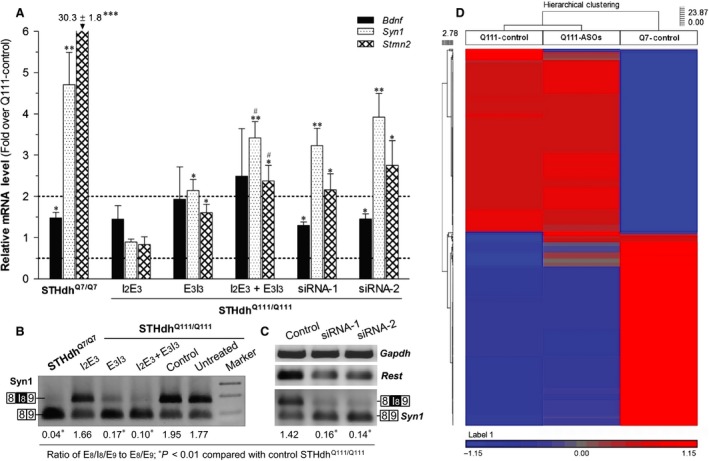
Comparison of REST‐controlled gene transcription between STHdh^Q7/Q7^ and STHdh^Q111/Q111^ cells with or without ASOs and siRNA treatment. (**A**) Transcriptional regulation of *Bdnf*,* Syn1* and *Stmn2* by ASOs and siRNAs. qRT‐PCR‐assayed mRNA levels of the genes were expressed as fold over the control STHdh^Q111/Q111^ and shown as Mean±SEM (*n* = 3). *P < 0.05, **P < 0.01, ***P < 0.001 compared with STHdh^Q111/Q111^ treated with a control oligo or non‐specific siRNA;. ^#^ *P* < 0.05 compared with the E_3_I_3_ group. (**B)** Syn1 mis‐splicing of in STHdh^Q7/Q7^ and STHdh^Q111/Q111^ cells with/‐out ASOs treatment. (**C)** Rescue of Syn1 mis‐splicing by Rest siRNAs. Note that amplicons shown for (**B**) and (**C**) were products of qRT‐PCR with Syn1‐E_8_F_1_/E_9_R_1,_ Rest‐E_3_F_1_/E_4_R_1_ and Gapdh‐F_3_/R_3_. Ratio of the two Syn1 variants with/‐out I_8_ was assessed by GeneTools software (Syngene) and averaged for three occasions. (**D)** Comparison of transcriptome between STHdh^Q7/Q7^ and STHdh^Q111/Q111^ with/‐out ASOs treatment. Three RNA samples (Q111‐Control, Q111‐ASOs and Q7‐Control) were transcriptionally profiled, and hierarchical clustering analysis of expression values was carried out using the genes generated by anova comparisons between the samples.

We then determined the effect of ASOs treatment on transcription of three well‐documented REST target genes, *Bdnf*,* Syn1* and *Stmn2* (also named *Scg10*), in STHdh^Q111/Q111^ cells. To evaluate the relevance of ASOs‐induced transcriptional regulation to altered Rest activity, we also examined regulation of the genes by two *Rest* siRNAs. As shown in Figure 3A, we found that (1) transcription of the three genes (especially *Stmn2*) was repressed in STHdh^Q111/Q111^ compared with STHdh^Q7/Q7^; (2) the ASOs treatment increased transcription of the genes depending on ∆E_3_ induction (I_2_E_3_ < E_3_I_3_ < I_2_E_3_ + E_3_I_3_); that is, ∆E_3_ exerts a dose‐dependent rescue of gene transcription; (3) ASOs‐induced transcriptional regulation was mimicked by two different siRNAs which reduced Rest mRNA expression by ~70% as assayed by qRT‐PCR with Rest‐E_3_F_1_/E_4_R_1_; and (4) compared with *Bdnf, Stmn2* and *Syn1* showed greater repression in STHdh^Q111/Q111^ and more derepression by ASOs/siRNAs.

Unexpectedly, we identified a new *Syn1* splice variant (*Syn1‐S*, GenBank Accession No. KJ174470) with I_8_ retention during verification of qRT‐PCR product for Syn1‐E_8_F_1_/E_9_R_1_. As shown in Figure 3B and C, we found that *Syn1‐S* was abundantly expressed in STHdh^Q111/Q111^ but barely in STHdh^Q7/Q7^, while its expression in STHdh^Q111/Q111^ was decreased by ASOs treatment in relation to ∆E_3_ induction and that the ASOs‐induced rescue of Syn1 mis‐splicing was mimicked by *Rest* siRNAs. Notably, I_8_ retention is predicted to introduce a pre‐mature stop codon and therefore predictive of a truncated N‐terminal Syn1‐S protein isoform missing partial domains and phosphorylation sites.

To determine whether additional genes were transcriptionally derepressed by the ASOs, we performed gene expression array to compare global transcription between three representative samples (Q111‐Control, Q111‐ASOs and Q7‐Control; GEO Accession No. GSE77194). As shown in Figure 3D and Table 2, global transcription differed significantly between STHdh^Q7/Q7^ and STHdh^Q111/Q111^ cells, while repression of numerous genes in STHdh^Q111/Q111^ was derepressed, at least in part, by ASOs treatment (I_2_E_3_ + E_3_I_3_). With a twofold change as the criteria for significance, the transcriptional data well supported the qRT‐PCR assayed *Stmn2* and *Syn1* expression change and extended ASOs‐induced transcriptional derepression to another 15 genes (*Sarm1, Nmnat2, Kcnk3, Stmn3, Gnptg, H2‐T23, Ppm1e, Fbxl16, Lrp11, Ina, Gdap1 l1, Unc13a, Vgf, Rundc3a* and *Rtn2*), of which *Nmnat2, Lrp11* and *Rtn2* were selectively confirmed by qRT‐PCR with additional samples (data not shown).

**Table 2:** Genes with more than twofold change in transcription induced by both mHtt and ASOs

Symbol	Fold change		Gene function
	Q7/Q111	ASOs/Ctrl	
*Sarm1*	2.1	2.2	Negative regulator of MYD88‐/TRIF‐dependent Toll‐like receptor signalling pathway which plays a pivotal role in activating axonal degeneration following injury; Also involved in immune response.
*Nmnat2*	6.3	2.2	Catalyses an essential step in NAD (NADP) biosynthetic pathway
*Kcnk3*	4.0	2.2	An outwardly rectifying channel sensitive to changes in extracellular pH
*Stmn2*	27.3	2.2	A member of the stathmin family of phosphoproteins; Regulator of microtubule stability and neuronal growth.
*Gnptg*	2.3	2.3	Catalyses the first step in synthesis of a mannose 6‐phosphate lysosomal recognition marker; Necessary for targeting of lysosomal hydrolases to the lysosome.
*Stmn3*	7.4	2.3	A member of the stathmin protein family which form a complex with tubulins; Involved in microtubule formation and function.
*H2‐T23*	2.3	2.3	Involved in immune response
*Ppm1e*	3.9	2.4	Dephosphorylates and inactivates multiple substrates including serine/threonine‐protein kinase 1, AMPK and the multifunctional calcium/calmodulin‐dependent protein kinases
*Fbxl16*	3.9	2.5	Functions in protein ubiquitination
*Gdap1 l1*	4.1	2.7	Likely functions in neuron differentiation; Associated with neuroblastoma.
*Syn1*	6.7	3.0	Plays a role in regulation of axonogenesis, synaptogenesis and neurotransmitter release
*Ina*	*2.4*	*3.1*	Facilitates axonal neurite elongation in neuroblastoma cells; Involved in morphogenesis of neurons
*Lrp11*	3.2	3.5	Involved in multicellular organismal response to stress
*Unc13a*	6.8	3.6	Binds to phorbol esters and diacylglycerol; Plays a role in neurotransmitter release at synapses
*Vgf*	6.4	3.7	Plays a role in maintenance of organismal energy balance and hippocampal synaptic activity
*Rundc3a*	9.3	3.9	Regulator of guanylate cyclase activity
*Rtn2*	10.0	6.1	Plays a role in generation of tubular endoplasmic reticulum and intracellular vesicular transport

Q7/Q111—relative mRNA expression in STHdh^Q7/Q7^ over STHdh^Q111/Q111^ (both were treated with a control oligo). ASOs/Ctrl—relative mRNA expression in ASOs‐treated STHdh^Q111/Q111^ over control STHdh^Q111/Q111^.

### ASOs‐induced protein expression change of Rest target genes in STHdh^Q111/Q111^ cells

**Figure 4:**
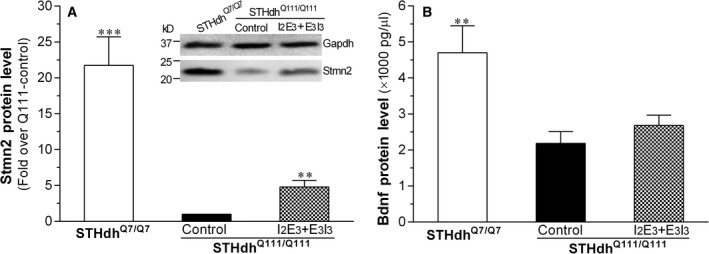
Comparison of Stmn2 (**A**) and Bdnf (**B**) protein expression between STHdh^Q7/Q7^ and STHdh^Q111/Q111^ cells with or without ASOs treatment. Western blotting and ELISA were performed to assay protein expression of Stmn2 and Bdnf, respectively. A representative Western blot for Stmn2 with Gapdh as a loading control was shown as an inset. Data are shown as Mean ± S.E.M. (*n* = 3). **P < 0.01, ***P < 0.001 compared with STHdh^Q111/Q111^ control by Student's *t‐*test.

We also performed Western blotting and ELISA to evaluate ASOs‐induced regulation of Stmn2 and Bdnf protein expression, respectively. As shown in Figure 4A and B, compared with STHdh^Q7/Q7^, STHdh^Q111/Q111^ expressed significantly lower levels of Stmn2 and BDNF, of which Stmn2 protein expression was significantly increased by ASOs treatment (I_2_E_3_ + E_3_I_3_), while there is a tendency for the BDNF protein expression to be increased by the ASOs.

## Discussion

As a major orchestrator of the cellular epigenome, REST governs the dynamic, context‐dependent expression of a huge gene network; however, it is poorly understood how REST function is environmentally tuned to moderate the dynamic, context‐dependent genome function. Emerging evidence indicates that *pre*‐mRNA splicing is environmentally regulated through epigenetic mechanisms 23, 24, suggesting a role of alternative splicing in environmentally tuned genome function fundamental to all aspects of cellular processes. In support of this notion, a shift from the full‐length REST to a truncated REST4, which is caused by inclusion of an extra exon (N_3c_) introducing a pre‐mature stop codon, contributes to neurogenesis—a process for neural stem/progenitor cells to differentiate into neurons 25. Recently, we demonstrated that *REST* undergoes extensive, context‐dependent alternative splicing, suggesting a major role of alternative splicing in environmental modulation of REST function; however, functionalities of most splicing events and mRNA variants are yet to be determined albeit somewhat predictable. Using specific ASOs and a cellular model of HD, our present study validates mechanism by which a common splicing (∆E_3_) modulates REST activity, as well as its potential as a therapeutic target for HD—a neurodegenerative disease associated with excessive nuclear REST.

**Figure 5:**
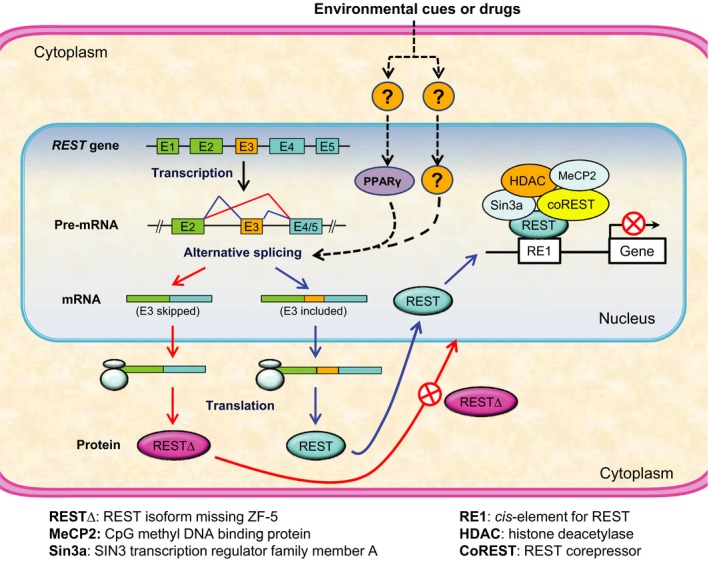
Illustration of ∆E_3_ as an endogenous, manipulable modulator of REST function. The splicing event ∆E_3_, which is common in human and non‐human primates, results in REST∆ protein isoform(s) missing ZF‐5 critical for nuclear targeting and therefore modulates nuclear REST levels and genome function. Note that (1) ∆E_3_ is modulated by PPARγ in a cell‐dependent manner, presumably through a *cis*‐element in E3; and (2) inclusion of E_5_ as the last exon, which is mutually exclusive to E_4_, was only observed in human but not rodents and non‐human primates 14.

ASOs are widely used for study of alternative splicing and may be utilized for gene therapy 26, 27, 28. Just as expected, the two ASOs targeting *Rest* E_3_ significantly induced ∆E_3_, reduced nuclear Rest and rescued neuronal gene expression in a cellular model of HD, while the ASOs‐induced transcriptional regulation was mimicked by siRNAs which considerably down‐regulate *Rest* mRNA expression, suggesting a causative link from ASOs‐induced ∆E_3_ to Rest activity. As ∆E_3_ does not occur naturally in rodents, our findings were unlikely confounded by endogenous ∆E_3_. So, as illustrated in Figure 5, this study provides evidence for ∆E_3_ as an endogenous modulator of REST activity as well as a potential therapeutic avenue for HD. As ∆E_3_ is modulated by pioglitazone 14—a highly selective PPARγ activator clinically used for type 2 diabetes but reportedly associated with increased risk of bladder cancer 29, it is possible that ∆E_3_ contributes, at least in part, to pharmacological actions of PPARγ and may thus have been inadvertently targeted for disease therapy. However, mechanisms involved in PPARγ regulation of gene expression are rather complex, and coinciding with the absence of natural ∆E_3_ in rodents, pioglitazone exerts no effect on ∆E_3_ in STHdh^Q111/Q111^ cells (data not shown), such that PPARγ ligands (*e.g*. pioglitazone) are not suitable for the study of ∆E_3_. As REST controls many cellular processes fundamental to normal physiology and disease aetiology, ∆E_3_ manipulation may have a broad range of clinical implications (*e.g*. cancer therapy and stem cell engineering). Notably, while the ASOs induced ∆E_3_ in both STHdh^Q7/Q7^ and STHdh^Q111/Q111^ cells, their effects on nuclear REST and gene transcription were only pronounced in STHdh^Q111/Q111^ but not in STHdh^Q7/Q7^ cells (data not shown), presumably due to a low level of basal nuclear Rest maintained by the normal nucleocytoplasmic shuttling in STHdh^Q7/Q7^ cells. Also, it should be pointed out that although both ∆E_3_ induction and RNAi down‐regulate Rest function, their effects are mediated by different mechanisms. While RNAi reduces globe expression of Rest isoforms including the full‐length Rest, ∆E_3_ induction does not affect total Rest expression but alters ratio of two types Rest isoforms discriminated by the capability of being transported into nucleus. Hence, a specific Rest isoform (*e.g*. the full‐length Rest) might be differentially influenced by the two approaches. For this reason, and considering the potential absence of correlation between expression levels of mRNA and protein 30, the effects of ∆E_3_ induction and RNAi on Rest target gene transcription may not be simply determined by the observed alteration in Rest expression. However, based on the ∆E_3_‐dependent effects of the ASOs on specific gene transcription (Fig. 3), a threshold along with a plateau likely exists for the transcriptional response to down‐regulation of Rest function. Presumably, once the plateau (*i.e*. maximum derepression) is achieved, further down‐regulation of Rest function will no longer increase transcriptional response, such may explain our observation of a similar transcriptional change yielded by ∆E_3_ induction and RNAi regardless of the differed downregulation of REST by the two approaches.

Although reduced BDNF expression has been documented in HD 31, it was reported that BDNF levels in human blood are not informative nor reliable as HD biomarkers 32. In accordance, we found that transcription of *Stmn2* and *Syn1,* but not *Bdnf*, was quite vulnerable to altered REST activity (*i.e*. up‐regulation by mHtt and downregulation by ASOs/siRNA), suggesting that *Stmn2* and *Syn1* may have advantage over *Bdnf* as potential HD biomarkers. Syn1 controls synapse function which is dysregulated in HD 33, 34, with abnormal phosphorylation of Syn1 being implicated in neurotransmission impairment in R6/2 HD mice 35. By identifying a REST‐controlled Syn1‐S isoform predictive of loss‐of‐function, this study provides a new mechanism underlying synapse dysfunction in HD. As alternative splicing is epigenetically regulated while REST orchestrates cellular epigenome, it is not surprising that pre‐mRNA splicing of *Syn1* (and probably other genes) is modulated by REST. Stmn2 functions in microtubule stability and neuronal growth, and given that decreased Stmn2 expression has been implicated in injury‐induced axonal degeneration 36, Down's syndrome 37, and AD 38, the striking repression of *Stmn2* in HD cells suggests a common Stmn2 dysfunction in a certain number of neurodegenerative diseases.

Besides *Bdnf*,* Stmn2* and *Syn1*, transcriptional profiling revealed ASOs‐induced derepression of other genes in STHdh^Q111/Q111^ cells. Despite the limited sample size, reliability of the transcriptional data was supported by several lines of evidence: (1) It is in good consistence with qRT‐PCR data for the tested genes; (2) bioinformatics indicates that almost all the transcriptionally regulated genes harbour at least one RE‐1 element; and (3) majority of the transcriptionally regulated genes are reportedly modulated by REST 39, 40, 41 and have been implicated in neurodegenerative diseases. For example, *Sarm1* and *Nmnat2* contribute to axonal degeneration—a critical, early event in neurodegenerative diseases 42, 43, while *Unc13a, Vgf, Rtn2, Lrp11* and *Rundc3a* have been implicated in amyotrophic lateral sclerosis 44, frontotemporal dementia 45, hereditary spastic paraplegias 46, and Parkinson's disease and AD 47, 48, 49. Thus, dysfunction of numerous specific genes is shared by neurodegenerative diseases, which may be explained by common pathogenic processes (*e.g*. axonal degeneration, endoplasmic reticulum stress and apoptosis) in such diseases. It should be pointed out that while only three samples were transcriptionally profiled, just a single dose and treatment duration of ASOs was examined in this study, making it possible that transcriptional regulation of some REST target genes cannot be observed in this study. Nevertheless, we demonstrate that repression of specific neuronal genes in STHdh^Q111/Q111^, which is presumably caused by mHtt‐induced excessive nuclear REST, can be rescued by ASOs‐induced ∆E_3_ which reduces nuclear REST.

While increased nuclear REST and its neurotoxicity in HD were well documented 8, 9, 10 and supported by this study, Lu *et al*. 7 recently reported reduced nuclear REST in AD and neuroprotection of REST in ageing brain. This discrepancy may reflect the complexity of REST function under distinct pathophysiological conditions; however, considering the extensive alternative *REST* splicing, variable assays of REST might be attributable. Of the 45 *REST* mRNA variants we previously identified, many are predictive of truncated protein products, of which REST4 is predicted by multiple mRNA variants 14, just like the case in rat 50. Notably, it is recently reported that (1) for E_2_‐lacking variants (*e.g*. E_1a_/E_3_/E_4_), an in‐frame AUG in E_3_ may initiate translation of a C‐terminal REST^C^ isoform (XP_005265817) 51; and (2) previously annotated noncoding RNAs with short ORF can encode small peptides 52, 53, such might be the case for numerous REST variants (*e.g*. JX896962, JX896965 and JX896967). Hence, REST protein isoforms might be much more complex than we expected; however, not all the predicted REST protein isoforms have been experimentally verified, and due to post‐translational modifications, they may not be observed as the predicted size by Western, making it challenging to determine whether an unexpected immunoreactive band is non‐specific or a REST isoform. Due to the extensive alternative *REST* splicing, it can be inferred that assay of REST by different primers/probes and antibodies may target different REST isoforms and therefore yield variable results. In support of this notion, the two anti‐REST antibodies used in this study yielded distinct immunostaining profiles (Fig. 2 and Fig. S1) and different immunoreactive bands in accordance with the manufacturer's instruction (data not shown). In the study of Lu *et al*. 7, only the full‐length REST was actually considered; however, qRT‐PCR with four primer sets targeting different exons of *REST* yielded different expression levels, providing evidence for the existence of alternative *REST* splicing 54. Notably, Lu *et al*. performed immunostaining with multiple anti‐REST antibodies without considering differences between the antibodies and disclosing detailed usage of the antibodies, making it possible that nuclear REST differences between the experimental groups might be confounded by biased usage of the antibodies for samples of different groups 54.

In summary, using specific ASO_S_ which induces a common alternative splicing (∆E_3_), we demonstrate that ∆E_3_ modulates nuclear REST and its gene regulation function in a cellular module of HD, and it thus represents a potential therapeutic target for HD. Our findings, which may extend to *in vivo* due to the ASOs‐induced ∆E_3_ in mouse primary neurons, highlight the role of alternative splicing in modulation of REST function and provide new insights into environmental regulation of genome function as well as the pathogenesis of HD.

## Conflict of interest

All of the authors do not have any financial disclosures to report.

## Supporting information


**Fig. S1** The effect of ASOs on ΔE_3_ in additional cells (A) and nuclear REST in RN46A cells (B).

**Fig. S2** Expanded view of immunofluorescence analysis of STHdh^Q7/Q7^ and STHdh^Q11/Q11^ cells with/‐out ASOs treatment.
